# A Low-Cost, High-Precision Vehicle Navigation System for Deep Urban Multipath Environment Using TDCP Measurements †

**DOI:** 10.3390/s20113254

**Published:** 2020-06-07

**Authors:** Jungbeom Kim, Minhuck Park, Yonghwan Bae, O-Jong Kim, Donguk Kim, Bugyeom Kim, Changdon Kee

**Affiliations:** 1 School of Mechanical and Aerospace Engineering and the Institute of Advanced Aerospace Technology, Seoul National University, Seoul 08826, Korea; magicromeo@snu.ac.kr (J.K.); kaito1125@snu.ac.kr (M.P.); yhbae1@snu.ac.kr (Y.B.); laywind0@snu.ac.kr (O.-J.K.); kbg7588@snu.ac.kr (B.K.); 2 Agency for Defense Development (ADD), Daejeon 34186, Korea; donguk319@add.re.kr

**Keywords:** GPS/INS, time-differenced carrier phase, urban environment, multipath

## Abstract

In this study, we developed a low-cost, high-precision vehicle navigation system for deep urban multipath environments using time-differenced carrier phase (TDCP) measurements. Although many studies are being conducted to navigate autonomous vehicles using the global positioning system (GPS), it is difficult to obtain accurate navigation solutions due to multipath errors in urban environments. Low-cost GPS receivers that determine the solution based on pseudorange measurements are vulnerable to multipath errors. We used carrier phase measurements that are more robust for multipath errors. Without correction information from reference stations, the limited information of a low-cost, single-frequency receiver makes it difficult to quickly and accurately determine integer ambiguity of carrier phase measurements. We used TDCP measurements to eliminate the need to determine integer ambiguity that is time-invariant and we combined TDCP-based GPS with an inertial navigation system to overcome deep urban multipath environments. Furthermore, we considered a cycle slip algorithm for its accuracy and a multi-constellation navigation system for its availability. The results of dynamic field tests in a deep urban area indicated that it could achieve horizontal accuracy of at the submeter level.

## 1. Introduction

Recently, global interest in autonomous vehicle navigation has increased, with extensive studies being conducted by many companies and research institutions. Most of these studies use light-based sensors—such as light detection and ragging (LiDAR) and cameras—for navigation [1,2,3,4], but there is a need for an alternative navigation system to handle cases where visibility is limited, such as in snow or fog [5,6]. The global positioning system (GPS) is widely employed to determine user position, velocity, and time (PVT) information based on signals broadcast through the GPS satellites in space. Many studies have been conducted on vehicle navigation using GPS [7,8,9,10]. Two kinds of measurements are employed to calculate position information using GPS receivers: pseudorange and carrier phase. Pseudorange measurements are difficult to use because of the extreme error of more than 100 m in a deep urban multipath environment. Therefore, most research is focused on carrier phase measurements, which are much more robust with a multipath error of less than 5 cm, using specificities of the received GPS signal such as shadow matching [11]. However, the method using the signal specificities is not able to provide precise accuracy for lane keeping. In addition, methods using carrier phase measurements, such as precise point positioning (PPP) and real-time kinematics (RTK), require an initial time and correction information from reference stations to quickly and accurately determine integer ambiguity [12,13,14]. It is more difficult to determine integer ambiguity for a moving land vehicle, which can be a major obstacle to real-time navigation. In addition, the price of a receiver providing PPP and RTK is generally high and can be a problem when considering the commercialization of autonomous vehicles, as most smartphones and car navigation systems are equipped with low-cost GPS receivers. 

In this study, we developed a low-cost, high-precision vehicle navigation system for deep urban multipath environments using time-differenced carrier phase measurements. In other words, this study suggests a navigation method using carrier phase measurements of low-cost, single-frequency GPS receivers, which are robust against multipath error. We utilized time differences on consecutive carrier phase measurements to eliminate the need to determine integer ambiguity that is time-invariant. These are called time-differenced carrier phase (TDCP) measurements and have previously been only used to improve the performance of altitude and velocity estimation [15,16,17]. No research has been conducted to identify the positioning accuracy by constructing a navigation system based on TDCP measurements in urban areas. The precise relative position is calculated based on TDCP measurements, and the absolute position is determined by accumulating the precise relative position based on the known initial position. When combined with the low-cost inertial navigation sensor (INS), a low-cost TDCP/INS system is constructed to complement the low sampling rates of GPS information, typically 1 Hz, and to detect occurrences of the cycle slip. The cycle slip detection algorithm, which must be included in the use of carrier phase measurements, utilizes an INS-based method [18]. In the implementation of the algorithm for cycle slip, the probability allocation is considered to reduce the occurrence of miss detection. The usual Kalman Filter (KF) model cannot be used because the TDCP measurements contain both the current and previous information, which violates the format of the usual KF model. Thus, we have already suggested a new optimal KF model to consider the correlated process and measurement noises in TDCP/INS systems [19]. Finally, a low-cost, high-precision vehicle navigation system for deep urban multipath environments is constructed using the TDCP/INS system including all algorithms such as the new optimal KF model and the cycle slip detection method [20]. To verify the performance of the proposed navigation system, actual dynamic vehicle tests were conducted in urban areas. The main advantage of the proposed navigation system is that it can be implemented only with low-cost equipment.

The remainder of this paper is organized as follows. Section 2 describes the theories underlying this study, Section 2.1 highlights the characteristics of urban environment and the detailed composition of the TDCP measurements, and Section 2.2 summarizes the navigation equations and the configuration of the proposed TDCP/INS system. Section 2.3 describes the detailed algorithm of cycle slip detection. Section 3 presents real dynamic experimental results, which confirm the positioning accuracy of the proposed system in urban multipath environments. Finally, Section 4 summarizes the main conclusions.

## 2. System Overview

In this section, the configuration and advantages of the TDCP measurements used in the proposed urban navigation system are described. The process to produce an optimal filter that combines TDCP measurements and INS is also presented, and the overall system configuration is introduced. Section 2.3 describes in detail the implementation of the cycle slip algorithm that addresses the failure of TDCP measurements.

### 2.1. Time-Differenced Carrier Phase (TDCP) Measurements

**Figure 1:**
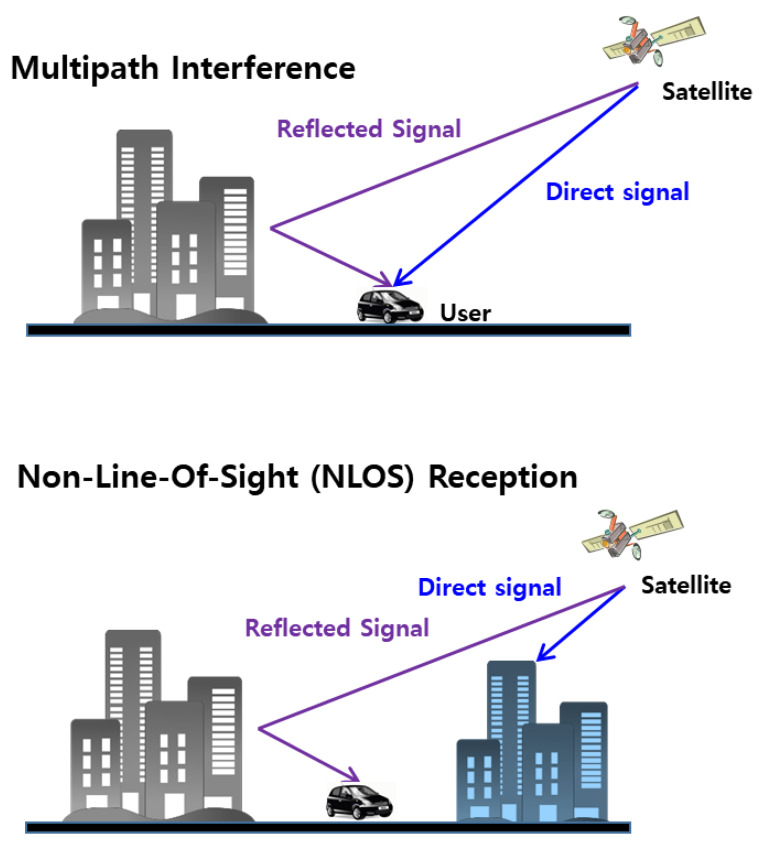
Multipath interference (where direct and reflected signals coexist) and non-line-of-sight (NLOS) reception (where only the reflected signal exists).

GPS signal-based navigation methods in urban multipath environments face huge challenges because buildings, traffic signs, and vehicles obstruct, reflect, and diffract signals [14]. As shown in Figure 1, the multipath phenomenon is classified into two cases—multipath interference and non-line of sight (NLOS) reception—which depend on whether direct signal from GPS satellites is received or not [21].

In the case of multipath interference, since the direct signal exists, the reflected signal causes an error while distorting the peak generated by the direct signal. Although it depends on the correlator design for each GPS receiver, the maximum error in pseudorange measurements is half of a ranging code chip (about 150 m) and the maximum error in carrier phase measurements is a quarter of a wavelength (about 4.76 cm) of L1 frequency (1575.42 MHz). In the case of NLOS reception, since there is no direct signal, the GPS receiver treats the reflected signal as a direct signal. Therefore, a ranging error equal to the path delay experienced by the reflected signal occurs in the pseudorange measurement. The corresponding ranging error in the carrier phase measurement is within half a wavelength of the pseudorange error (modulo one carrier cycle) as the path delay is the same [22]. In both cases, the multipath error in the carrier phase measurement is much smaller than that of the pseudorange measurement. The pseudorange (ρ) and carrier phase (ϕ) measurements can be expressed as follows:

(1) $$\rho_{u}^{i}=\left({\bar{r}}^{i}+\delta {\bar{r}}^{i}-{\bar{r}}_{u}\right)\cdot {\bar{e}}_{u}^{i}+B_{u}+T_{u}^{i}+I_{u}^{i}-b^{i}+M_{u}^{i}+\varepsilon_{\rho ,u}^{i}$$

(2) $$\phi_{u}^{i}=\left({\bar{r}}^{i}+\delta {\bar{r}}^{i}-{\bar{r}}_{u}\right)\cdot {\bar{e}}_{u}^{i}+B_{u}+T_{u}^{i}-I_{u}^{i}-b^{i}+m_{u}^{i}+\varepsilon_{\phi ,u}^{i}+\lambda N_{u}^{i}$$

The superscript *i* denotes the *i*th satellite, and the subscript *u* denotes the user. $\bar{r},\bar{e},N,\lambda$ are the position, line-of-sight vector, integer ambiguity, and wavelength of L1 frequency, respectively. The error variables $b,B,I,T,\delta \bar{r},M,m,\varepsilon_{\rho }$, and $\varepsilon_{\phi }$ are satellite clock bias, receiver clock bias, ionospheric delay, tropospheric delay, broadcasted orbit error, multipath error in pseudorange measurement, multipath error in carrier phase measurement, noise of pseudorange measurement, and noise of the carrier phase measurement, respectively. As described above, the multipath error in pseudorange measurements (M) has a much larger size than the multipath error in carrier phase measurements (m), which makes it difficult to distinguish where the dynamic land vehicle is located in urban areas based on the low-cost L1 single-frequency GPS receiver. 

**Figure 2:**
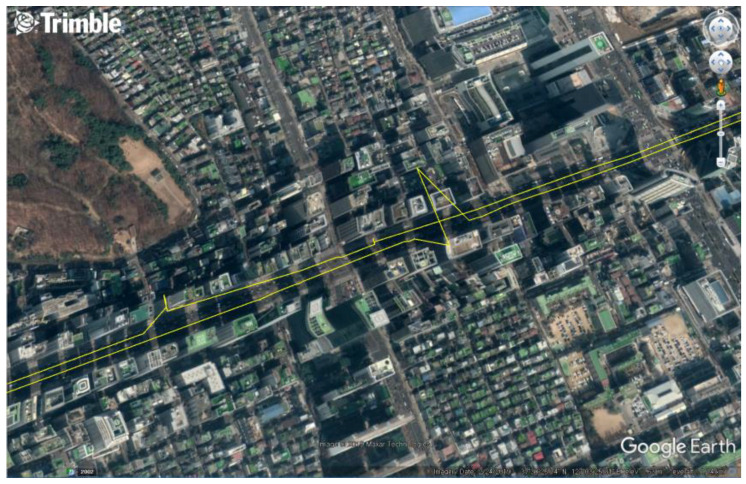
Trajectory of a dynamic land vehicle in an urban multipath area.

A representative expensive dual-frequency GPS receiver (Trimble NetR9), was used to collect the data from a land vehicle driving a straight line in an urban area to identify the effects of multipath errors. A yellow trajectory calculated with reference station data using Trimble business center (TBC) post-processing software can be seen affected by the multipath error, as shown in Figure 2. 

Generally, precise navigation based on carrier phase measurements is possible using methods such as RTK and PPP based on data from an expensive dual-frequency GPS receiver with reference station data. However, the error shown in Figure 2 appears to be caused using pseudorange measurements contaminated by multipath errors when the integer ambiguity cannot be determined due to a drop in the quality of the carrier phase measurement in the deep urban area.

**Figure 3:**
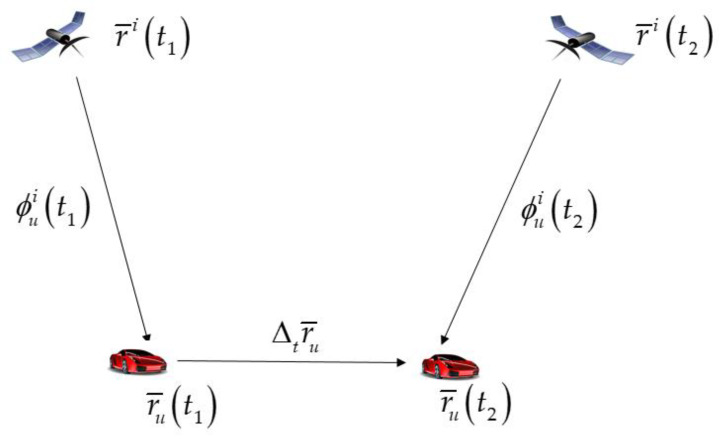
Consecutive carrier phase measurements at time epochs $t_{1}$ and $t_{2}$.

In this study, an urban navigation system is constructed using carrier phase measurements with an extremely low influence from multipath error. Generally, it is quite difficult for dynamic users in the nondifferential mode to resolve integer ambiguity. However, integer ambiguity is a constant that can be eliminated by taking the difference of two consecutive GPS epochs. The consecutive carrier-phase measurements are shown in Figure 3. 

By subtracting the consecutive carrier phase measurements at time epochs $t_{1}$ and $t_{2}$, the TDCP measurement can be obtained as follows:

(3) $$\begin{array}{ll} \Delta_{t}\phi_{u}^{i} & =\phi_{u}^{i}\left(t_{2}\right)-\phi_{u}^{i}\left(t_{1}\right)\\ & =\left\{{\bar{r}}^{i}\left(t_{2}\right)-{\bar{r}}_{u}\left(t_{2}\right)\right\}\cdot {\bar{e}}_{u}^{i}\left(t_{2}\right)-\left\{{\bar{r}}^{i}\left(t_{1}\right)-{\bar{r}}_{u}\left(t_{1}\right)\right\}\cdot {\bar{e}}_{u}^{i}\left(t_{1}\right)\\ & \;\;+\left\{\delta {\bar{r}}^{i}\left(t_{2}\right)\cdot {\bar{e}}_{u}^{i}\left(t_{2}\right)-\delta {\bar{r}}^{i}\left(t_{1}\right)\cdot {\bar{e}}_{u}^{i}\left(t_{1}\right)\right\}+\Delta_{t}B_{u}+\Delta_{t}T_{u}^{i}-\Delta_{t}I_{u}^{i}-\Delta_{t}b^{i}+\Delta_{t}m^{i}+\Delta_{t}\varepsilon_{u}^{i}\\ & ={\bar{r}}^{i}\left(t_{2}\right)\cdot {\bar{e}}_{u}^{i}\left(t_{2}\right)-{\bar{r}}^{i}\left(t_{1}\right)\cdot {\bar{e}}_{u}^{i}\left(t_{1}\right)-\Delta_{t}{\bar{r}}_{u}\cdot {\bar{e}}_{u}^{i}\left(t_{2}\right)-{\bar{r}}_{u}\left(t_{1}\right)\cdot \Delta_{t}{\bar{e}}_{u}^{i}\\ & \;\;+\left\{\delta {\bar{r}}^{i}\left(t_{2}\right)\cdot {\bar{e}}_{u}^{i}\left(t_{2}\right)-\delta {\bar{r}}^{i}\left(t_{1}\right)\cdot {\bar{e}}_{u}^{i}\left(t_{1}\right)\right\}+\Delta_{t}B_{u}+\Delta_{t}T_{u}^{i}-\Delta_{t}I_{u}^{i}-\Delta_{t}b^{i}+\Delta_{t}m^{i}+\Delta_{t}\varepsilon_{u}^{i} \end{array}$$

where N has been eliminated through time differencing. $\Delta_{t}$ is the time difference operator and signifies the change during the period $t_{2}-t_{1}$. To calculate the relative position $\Delta_{t}{\bar{r}}_{u}$ using the least-squares method, Equation (3) can be modified as follows with m visible satellites in matrix form:

(4) $$\begin{array}{l} \Delta_{t}{\bar{r}}_{u}\cdot {\bar{e}}_{u}^{i}\left(t_{2}\right)-\Delta_{t}B_{u}={\bar{r}}^{i}\left(t_{2}\right)\cdot {\bar{e}}_{u}^{i}\left(t_{2}\right)-{\bar{r}}^{i}\left(t_{1}\right)\cdot {\bar{e}}_{u}^{i}\left(t_{1}\right)-{\bar{r}}_{u}\left(t_{1}\right)\cdot \Delta_{t}{\bar{e}}_{u}^{i}-\Delta_{t}\phi_{u}^{i}+\Delta_{t}E_{u}^{i}\\ \left[\begin{array}{cc} {\bar{e}}_{u}^{1}(t_{2}) & -1\\ \vdots & \vdots \\ {\bar{e}}_{u}^{m}(t_{2}) & -1 \end{array}\right]\left(\begin{array}{c} \Delta_{t}{\bar{r}}_{u}\\ \Delta_{t}B_{u} \end{array}\right)=\left[\begin{array}{c} {\bar{r}}^{1}\left(t_{2}\right)\cdot {\bar{e}}_{u}^{1}\left(t_{2}\right)-{\bar{r}}^{1}\left(t_{1}\right)\cdot {\bar{e}}_{u}^{1}\left(t_{1}\right)-{\bar{r}}_{u}\left(t_{1}\right)\cdot \Delta_{t}{\bar{e}}_{u}^{1}-\Delta_{t}\phi_{u}^{1}+\Delta_{t}E_{u}^{1}\\ \vdots \\ {\bar{r}}^{m}\left(t_{2}\right)\cdot {\bar{e}}_{u}^{m}\left(t_{2}\right)-{\bar{r}}^{m}\left(t_{1}\right)\cdot {\bar{e}}_{u}^{m}\left(t_{1}\right)-{\bar{r}}_{u}\left(t_{1}\right)\cdot \Delta_{t}{\bar{e}}_{u}^{m}-\Delta_{t}\phi_{u}^{m}+\Delta_{t}E_{u}^{m} \end{array}\right] \end{array}$$

where $\Delta_{t}E_{u}^{i}=\Delta_{t}T_{u}^{i}-\Delta_{t}I_{u}^{i}-\Delta_{t}b^{i}+\Delta_{t}m^{i}+\Delta_{t}\varepsilon_{u}^{i}+\left\{\delta {\bar{r}}^{i}\left(t_{2}\right)\cdot {\bar{e}}_{u}^{i}\left(t_{2}\right)-\delta {\bar{r}}^{i}\left(t_{1}\right)\cdot {\bar{e}}_{u}^{i}\left(t_{1}\right)\right\}$ is the total change of the GPS error sources and noise. Equation (4) can also be expressed more simply by using the satellite difference so as not to estimate $\Delta_{t}B_{u}$, as follows:

(5) $$\begin{array}{l} \Delta_{t}{\bar{r}}_{u}\cdot^{i}\nabla^{R}{\bar{e}}_{u}^{}\left(t_{2}\right){=}^{i}\nabla^{R}\bar{r}\left(t_{2}\right)\cdot {\bar{e}}_{u}^{}\left(t_{2}\right){-}^{i}\nabla^{R}\bar{r}\left(t_{1}\right)\cdot {\bar{e}}_{u}^{}\left(t_{1}\right)-{\bar{r}}_{u}\left(t_{1}\right)\cdot^{i}\nabla^{R}\Delta_{t}{\bar{e}}_{u}^{}{-}^{i}\nabla^{R}\Delta_{t}\phi_{u}^{}{+}^{i}\nabla^{R}\Delta_{t}E_{u}^{}\\ \begin{array}{c} \left[\begin{array}{c} {}^{1}\nabla^{R}{\bar{e}}_{u}^{}(t_{2})\\ \vdots \\ {}^{m-1}\nabla^{R}{\bar{e}}_{u}^{}(t_{2}) \end{array}\right]\left(\Delta_{t}{\bar{r}}_{u}\right)\\ =\left[\begin{array}{c} {}^{1}\nabla^{R}\bar{r}\left(t_{2}\right)\cdot {\bar{e}}_{u}^{}\left(t_{2}\right){-}^{1}\nabla^{R}\bar{r}\left(t_{1}\right)\cdot {\bar{e}}_{u}^{}\left(t_{1}\right)-{\bar{r}}_{u}\left(t_{1}\right)\cdot^{1}\nabla^{R}\Delta_{t}{\bar{e}}_{u}^{}{-}^{1}\nabla^{R}\Delta_{t}\phi_{u}^{}{+}^{1}\nabla^{R}\Delta_{t}E_{u}^{}\\ \vdots \\ {}^{m-1}\nabla^{R}\bar{r}\left(t_{2}\right)\cdot {\bar{e}}_{u}^{}\left(t_{2}\right){-}^{m-1}\nabla^{R}\bar{r}\left(t_{1}\right)\cdot {\bar{e}}_{u}^{}\left(t_{1}\right)-{\bar{r}}_{u}\left(t_{1}\right)\cdot^{m-1}\nabla^{R}\Delta_{t}{\bar{e}}_{u}^{}{-}^{m-1}\nabla^{R}\Delta_{t}\phi_{u}^{}{+}^{m-1}\nabla^{R}\Delta_{t}E_{u}^{} \end{array}\right] \end{array} \end{array}$$

The superscript R denotes the reference satellite, which is typically defined as the highest elevated satellite among all of the satellites because it could have the smallest error factors and noise, and is most likely to receive direct signal from the GPS satellite, in the satellite difference represented by ∇. 

Equation (5) can be expressed in the Hx=z format, and $\Delta_{t}{\bar{r}}_{u}$ can be determined as follows [19]:

(6) $$\Delta_{t}{\bar{r}}_{u}={\left(H^{T}H\right)}^{-1}H^{T}z$$

The relative position calculated as Equation (6) by TDCP measurements has a millimeter-level accuracy, which is known to be more accurate than the relative position of centimeter-level accuracy obtained by Doppler measurement [23,24]. 

### 2.2. TDCP/INS Integrated Navigation System

A system of autonomous vehicles generally has the properties of nonlinear systems. Thus, concepts of extended KF (EKF) are used to integrate TDCP with INS. The process and measurement models of the basic EKF can be defined as follows:

(7) $$\begin{array}{l} \delta x_{k+1}=\Phi_{k}\delta x_{k}+w_{k}\\ \delta z_{k}=H_{k}\delta x_{k}+v_{k} \end{array}$$

where δ denotes the residual error of variable. The subscripts k+1 and k denote the *k+1* and *k*th time epochs, respectively; The terms Φ,  H,  w, and v are the state transition matrix, observation matrix, process noise, and measurement noise, respectively. Generally, both noise terms are not cross-correlated and have the following characteristics:

(8) $$\begin{array}{l} E\left[w_{k}w_{k}^{T}\right]=\{\begin{array}{cc} Q_{k}, & i=k\\ 0, & i\neq k \end{array}\\ E\left[v_{k}v_{k}^{T}\right]=\{\begin{array}{cc} R_{k}, & i=k\\ 0, & i\neq k \end{array} \end{array}$$

However, the integration is challenging following the basic EKF models because the TDCP measurement includes both current and previous position information, which violates the basic assumption of the KF that only current information should be used. Therefore, to use the TDCP measurement, the new measurement update model of EKF must be composed using the concept of a delayed-state filter [25]. 

The new measurement model consists of information from two consecutive epochs and can be expressed as:

(9) $$\delta z_{k}=H_{k}\delta x_{k}+J_{k}\delta x_{k-1}+v_{k}$$

where J is another observation matrix, which is similar to H. The definitions of J and H will be provided later in detail. From Equation (7), it is possible to consider backward time propagation as follows:

(10) $$\delta x_{k-1}=\Phi_{k-1}^{-1}\delta x_{k}-\Phi_{k-1}^{-1}w_{k-1}$$

Equation (10) can be substituted into Equation (9), and a new measurement model can be derived for TDCP measurements as follows:

(11) $$\begin{array}{ll} \delta z_{k} & =H_{k}\delta x_{k}+J_{k}\left(\Phi_{k-1}^{-1}\delta x_{k}-\Phi_{k-1}^{-1}w_{k-1}\right)+v_{k}\\ & =\left(H_{k}+J_{k}\Phi_{k-1}^{-1}\right)\delta x_{k}+\left(-J_{k}\Phi_{k-1}^{-1}w_{k-1}+v_{k}\right)\\ & ≜{H^{\prime }}_{k}\delta x_{k}+{v^{\prime }}_{k} \end{array}$$

As shown in Equation (11), the new quantities ${H^{\prime }}_{k}$ and ${v^{\prime }}_{k}$ are obtained, and it is necessary to derive new covariance matrices associated with them, as follows:

(12) $$\begin{array}{ll} {R^{\prime }}_{k} & =E\left[{v^{\prime }}_{k}{v^{\prime }}_{k}{}^{T}\right]\\ & =E\left[\left(-J_{k}\Phi_{k-1}^{-1}w_{k-1}+v_{k}\right){\left(-J_{k}\Phi_{k-1}^{-1}w_{k-1}+v_{k}\right)}^{T}\right]\\ & =J_{k}\Phi_{k-1}^{-1}Q_{k-1}\Phi_{k-1}^{-1T}J_{k}^{T}+R_{k} \end{array}$$

(13) $$\begin{array}{ll} C_{k} & =E\left[w_{k-1}{v^{\prime }}_{k}{}^{T}\right]\\ & =E\left[w_{k-1}{\left(-J_{k}\Phi_{k-1}^{-1}w_{k-1}+v_{k}\right)}^{T}\right]\\ & =-Q_{k}\Phi_{k}^{-1T}J_{k+1}^{T} \end{array}$$

The new error covariance matrix $P_{k}^{+}$ and Kalman gain $K_{k}$, taking into account the changed new covariance matrices above from Equations (12) and (13), are given by:

(14) $$\begin{array}{l} P_{k}^{+}=\left(I-K_{k}{H^{\prime }}_{k}\right)P_{k}^{-}{\left(I-K_{k}{H^{\prime }}_{k}\right)}^{T}+K_{k}{R^{\prime }}_{k}K_{k}^{T}\\ \;\;\;\;\;\;\;\;\;\;\;\;-\left(I-K_{k}{H^{\prime }}_{k}\right)C_{k}K_{k}^{T}-K_{k}C_{k}^{T}{\left(I-K_{k}{H^{\prime }}_{k}\right)}^{T} \end{array}$$

(15) $$K_{k}=\left(P_{k}^{-}{H^{\prime }}_{k}^{T}+C_{k}\right){\left[{H^{\prime }}_{k}P_{k}^{-}{H^{\prime }}_{k}^{T}+{R^{\prime }}_{k}+{H^{\prime }}_{k}C_{k}+C_{k}^{T}{H^{\prime }}_{k}^{T}\right]}^{-1}$$

The superscript ^–^ denotes the states before measurement update. The superscript ^+^ denotes after measurement update. The time update model, which is the same as that for a basic EKF, can be summarized as follows:

(16) $$\begin{array}{l} \delta {\hat{x}}_{k+1}^{-}=\Phi_{k}\delta {\hat{x}}_{k}^{+}\\ P_{k+1}^{-}=\Phi_{k}P_{k}^{+}\Phi_{k}^{T}+Q_{k} \end{array}$$

The method of the construction of a more detailed TDCP/INS model can be found in a previous study [19]. 

**Figure 4:**
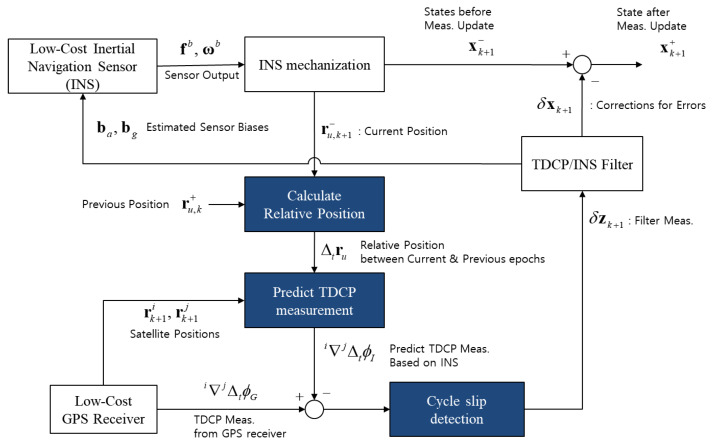
Configuration of the time-differenced carrier phase/inertial navigation sensor (TDCP/INS) navigation system.

Figure 4 shows the configuration of the TDCP/INS navigation system. It consists of only two sensors: a low-cost INS and a GPS receiver to collect TDCP measurements. The system determines the precise absolute position by accumulating an estimated precise relative position based on the TDCP/INS navigation system starting from a known initial position. The structure of the TDCP/INS navigation system is described in detail as follows. Firstly, the relative position ($\Delta_{t}r_{u}$) is calculated based on INS. Secondly, the system predicts the TDCP measurements (${}^{i}\nabla^{j}\Delta_{t}\phi_{I}$) based on the calculated relative position. The predicted TDCP measurements are then used to detect the cycle slip of the measured TDCP (${}^{i}\nabla^{j}\Delta_{t}\phi_{G}$). Lastly, based on the clear TDCP measurements after cycle slip detection, the TDCP/INS filter estimates the error components of states ($x_{k+1}^{-}$) and corrects them to maintain an accurate navigation performance. 

The perturbation equation for INS can be derived with respect to different types of coordinate frames. In this study, the Earth-centered Earth-fixed (ECEF) frame was utilized, and the state vectors of the system consist of 15 elements, which are as follows:

(17) $$\delta x=\left[\begin{array}{ccccc} \delta P^{e} & \delta V^{e} & \delta \psi^{e} & \delta b_{a}^{e} & \delta b_{g}^{e} \end{array}\right]$$

The superscript e denotes states in the ECEF frame. The states $\delta P^{e},\delta V^{e}$, and $\delta \psi^{e}$ are position, velocity, and orientation errors, respectively; $\delta b_{a}$ and $\delta b_{g}$ are accelerometer and gyroscope bias errors, respectively. The detailed INS error equations are found in [26]. The measurement of the tightly coupled KF filter consists of the satellite-differenced TDCP measurements. The observation matrices are defined as follows:

(18) $$\begin{array}{l} H_{k}=\left[\begin{array}{ccccc} {-}^{1}\nabla^{R}{\bar{e}}_{u,k}^{T} & 0_{3\times 1} & 0_{3\times 1} & 0_{3\times 1} & 0_{3\times 1}\\ \vdots & \vdots & \vdots & \vdots & \vdots \\ {-}^{n-1}\nabla^{R}{\bar{e}}_{u,k}^{T} & 0_{3\times 1} & 0_{3\times 1} & 0_{3\times 1} & 0_{3\times 1} \end{array}\right]\\ J_{k}=-H_{k} \end{array}$$

### 2.3. Cycle Slip Detection Algorithm

**Figure 5:**
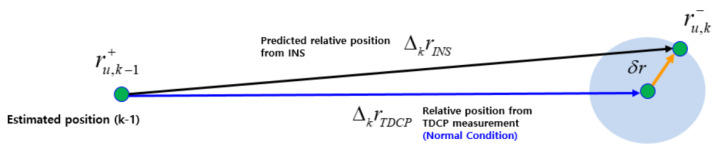
Relationship between INS and TDCP-based relative positions (normal condition).

In a navigation system using the carrier phase measurement, a large positioning error may be caused by cycle slip, which is a discontinuity of the integer ambiguity in the measured carrier phase [27,28]. In real-time kinematic applications, especially in an urban environment, GPS signals could be lost temporarily because of various disturbing factors such as blockage by buildings, trees, and bridges and by vehicle dynamics. Thus, an algorithm to deal with the cycle slip is necessary. Although algorithms related to the cycle slip detection exist in various ways using dual-frequency measurements, in this study, the INS-based approach was utilized with only limited low-cost, single-frequency GPS receiver information. Figure 5 shows the relationship of relative positions between consecutive k−1 and kth, estimated based on INS and TDCP. In normal conditions, the kth predicted absolute position ($r_{u,k}^{-}$) is determined by the relative position estimated based on the INS ($\Delta_{k}r_{INS}$) at the k−1 estimated position ($r_{u,k-1}^{+}$), where $\Delta_{k}$ is the time difference operator and signifies the change between consecutive k−1 and kth epochs. Updates to the EKF measurement for TDCP/INS are performed to correct the INS propagation error by comparing the difference (δr) with the relative positions estimated by the INS and TDCP. Here, the blue circle in Figure 5 is the margin of error that the position predicted by INS can have. This can be calculated from the sensor specification of the INS [18]. 

**Figure 6:**
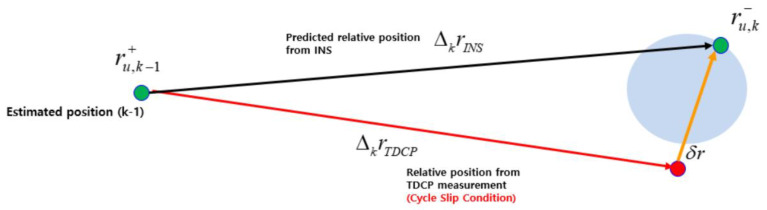
Relationship between INS and TDCP-based relative positions (cycle slip condition).

The INS-based cycle slip detection method in this study used the above relationship of relative positions inversely as shown in Figure 6. As described above, it is possible to predict the margin of error by INS as the blue circle area in Figure 6, and it is also possible to determine whether the TDCP measurement is normal or abnormal based on this area. Specifically, if a cycle slip occurs, the position based on TDCP measurement will be found out of the blue area, as shown by the red line and point, so the cycle slip can be detected. 

It can be summarized as follows. Firstly, differentiating the carrier phase measurement of *i*th satellite with respect to the reference satellite and time, we can obtain a double-differenced measurement as follows:

(19) $${}^{i}\nabla^{r}\Delta_{t}\phi_{u}^{}{=}^{i}\nabla^{r}\Delta_{t}d_{u}^{}{+}^{i}\nabla^{r}\Delta_{t}\lambda N_{u}^{}{+}^{i}\nabla^{r}\Delta_{t}E$$

The superscript r denotes the reference satellite. Equation (19) consists of actual travel distance (d), integer ambiguity and residual error (E). The ${}^{i}\nabla^{r}\Delta_{t}E\equiv^{i}\nabla^{r}\Delta_{t}I_{u}{+}^{i}\nabla^{r}\Delta_{t}T_{u}{+}^{i}\nabla^{r}\Delta_{t}\varepsilon_{\phi ,u}$ is the residual error after time difference, and it can be ignored because it is much smaller compared to 1 cycle slip (about 20 cm) [29]. Furthermore, since the actual travel distance term can be estimated based on the INS, the cycle lip can be detected using the difference between measured and estimated values. We set it as the monitoring value (M) for cycle slip detection.

(20) $$M_{k+1}^{i}{=}^{i}\nabla^{r}\Delta_{t}\phi_{u}{-}^{i}\nabla^{r}\Delta_{t}\phi_{I}$$

The subscripts u and I denote measured and estimated values. In this study, compensation of cycle slip is not performed, and the measurement is just removed to calculate the navigation solution if a cycle slip is detected.

When designing the cycle slip detection algorithm, the false alarm (FA) and miss detection (MD) probabilities should be considered. In general, MD is more important than FA because MD causes degradation of positioning accuracy. Thus, we designed the cycle slip algorithm focusing on the MD probability. We fixed MD probability for all satellites considering a worst-case scenario [30]. 

**Figure 7:**
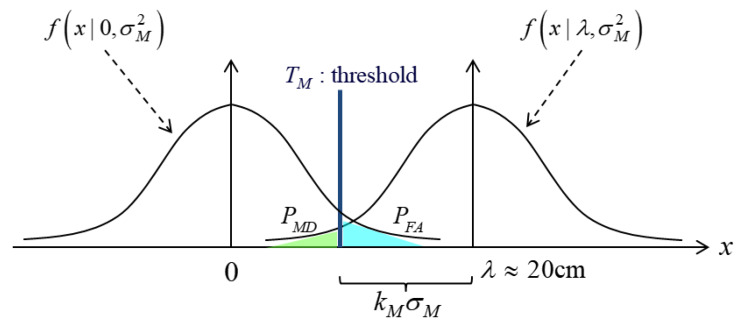
Relationship of probabilities between false alarm (FA) and miss detection (MD).

Figure 7 shows the relationship between FA and MD probabilities. The detection threshold ($T_{M}$) for 1 cycle slip is calculated as follows:

(21) $$T_{M}=\lambda -k_{M}\sigma_{M}$$

where $P_{MD}=2\times 10^{-6},k_{M}=4.75$, by referring to a previous study [30]. The $\sigma_{M}^{2}$ is a standard deviation of the monitoring value, and it can be summarized as follows:

(22) $$\sigma_{M}^{2}=\mathrm{var}\left({}^{i}\nabla^{r}{\bar{e}}_{}\cdot \delta \;\Delta_{t}r_{u}\right)+\sigma_{\nabla \Delta_{t}E}^{2}$$

where $\bar{e}$ is a line-of-sight vector, $\delta \;\Delta_{t}r_{u}$ is an estimation error due to INS, and $\sigma_{\nabla \Delta_{t}E}^{2}$ is the standard deviation of the residual error. In deep urban areas, $\sigma_{M}^{2}$ can be quite large due to signal tracking and multipath errors. In this case, it causes frequent FA and reduces system availability. Therefore, to improve the availability, we utilized multi-constellation and ensured that the navigation system did not estimate differences in the system clock, which does not change by more than 1 m during a day [31]. Finally, we can construct a robust cycle slip algorithm for deep urban areas while maintaining the probability of MD and considering the worst case.

## 3. Dynamic Test in Deep Urban Multipath Environment

### 3.1. Test Environment

**Figure 8:**
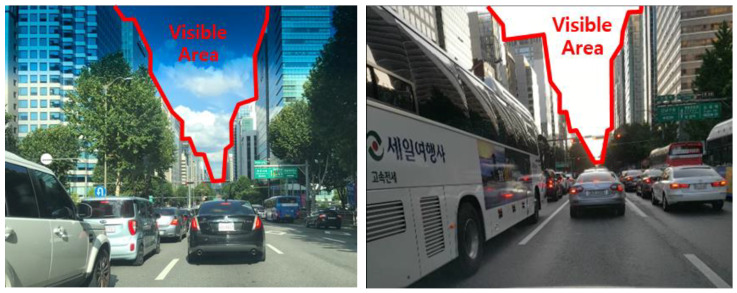
Teheran road in Seoul (dynamic test environment).

To confirm the performance of the TDCP/INS navigation system, we conducted a vehicle-based dynamic experiment in a deep urban multipath area. The data were collected while driving a land vehicle on Teheran road, a highly urbanized area of Seoul, as shown in Figure 8.

We used ADIS16405IMU as the inertial measurement unit (IMU) and two low-cost, single-frequency Ublox M8T GPS receivers to collect measurements from four constellations: GPS in the United States of America (USA), the global navigation satellite system (GLONASS) in Russia, BeiDou in China, and Galileo in Europe. This is because M8T cannot collect all satellite data at the same time for reasons including insufficient channels and front-ends. The two M8T receivers were divided into #1 M8T (receiving GPS and BeiDou) and #2 M8T (receiving GPS, GLONASS, and Galileo). Here, the problem of the receiver clock error caused by using two different receivers was eliminated by the satellite difference effect of using the same reference satellite. The GPS and IMU output rates were set to 1 and 100 Hz. Any correction information such as PPP and error models were not used except for the wide-area augmentation system (WAAS) tropospheric delay model.

**Figure 9:**
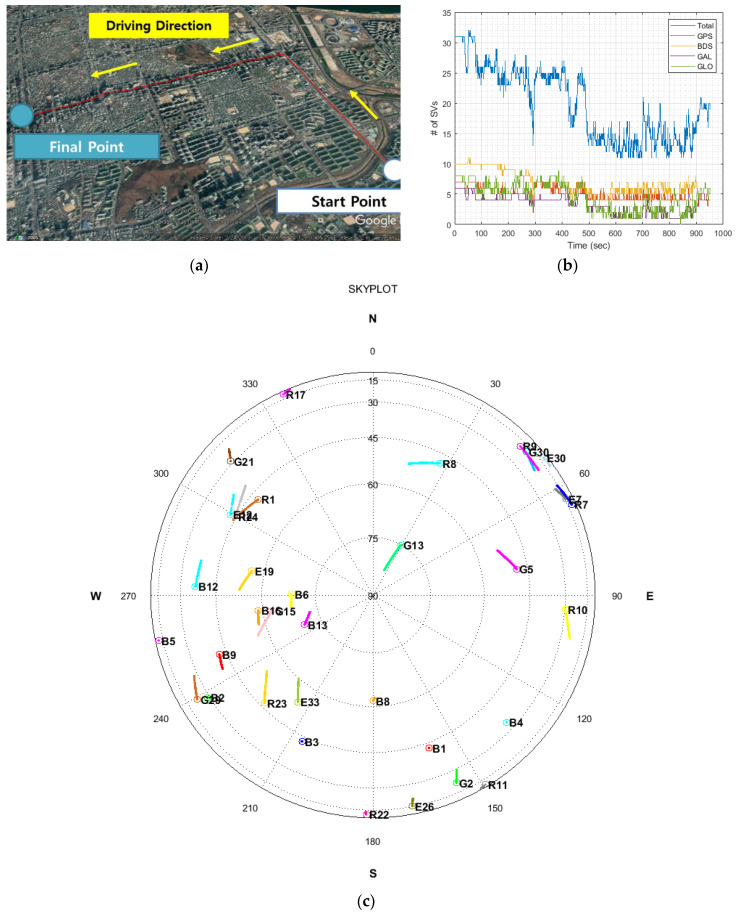
(**a**) Tracked trajectory; (**b**) number of visible satellites; (**c**) sky plot.

This dynamic experiment was performed for 15 min on April 12, 2019. Figure 9a–c shows the tracked trajectory, number of satellites, and sky plot during the experiment, respectively. In Figure 9a, the red line shows the trajectory of driving the land vehicle in the deep urban multipath area. In the sky plot, G, R, B, and E represent the GPS satellite, GLONASS, BeiDou, and Galileo, respectively. As can be seen from Figure 9b, the number of visible satellites decreased when the vehicle was driven to the extreme urban area more than the early stage of driving. 

We collected the experimental data and analyzed the results by post-processing. At this time, an accurate reference trajectory was needed to analyze the performance of the proposed TDCP/INS navigation system precisely. For the reference trajectory, dual-frequency, multi constellation (GPS, GLONASS, BeiDou, and Galileo) GPS measurements received from Novatel PwrPak7 and INS data from SPAN-CPT were processed using a Novatel post-processing program called Waypoint version 8.8 (the latest version) [32]. 

### 3.2. Test Results

**Figure 10:**
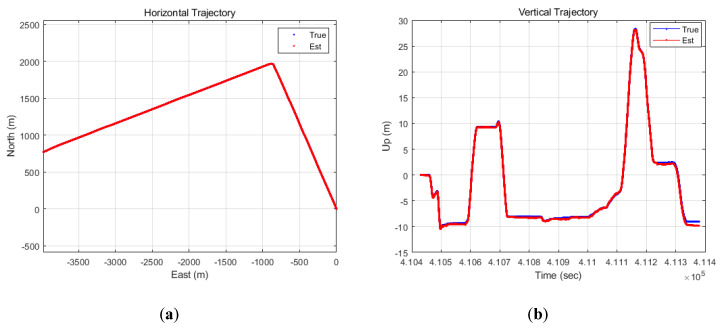
(**a**) Horizontal trajectory; (**b**) vertical trajectory (TDCP/INS).

Figure 10 shows the horizontal and vertical trajectories. The blue points indicate the reference trajectory and red points indicate the estimated trajectory of the proposed TDCP/INS navigation system. When comparing the entire trajectories, the magnitude of the error could not be easily confirmed.

**Figure 11:**
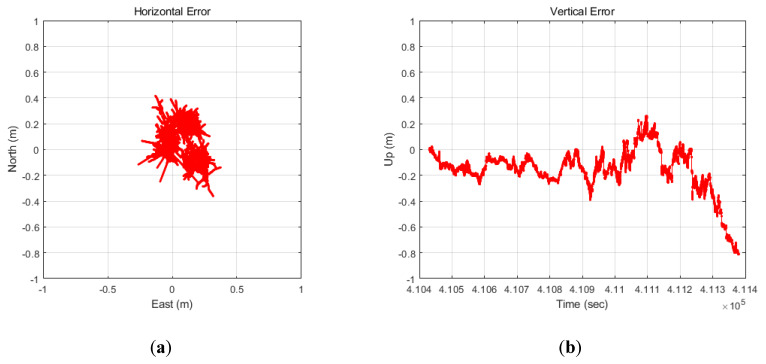
(**a**) Horizontal error; (**b**) vertical error (TDCP/INS).

Figure 11 shows the horizontal and vertical errors. In this study, we assumed that the initial position is known exactly. At this time, the results confirmed that the position errors, accumulated through the TDCP/INS for 15 min relative to the initial position, were under 1 m in both the horizontal and vertical axes. 

**Figure 12:**
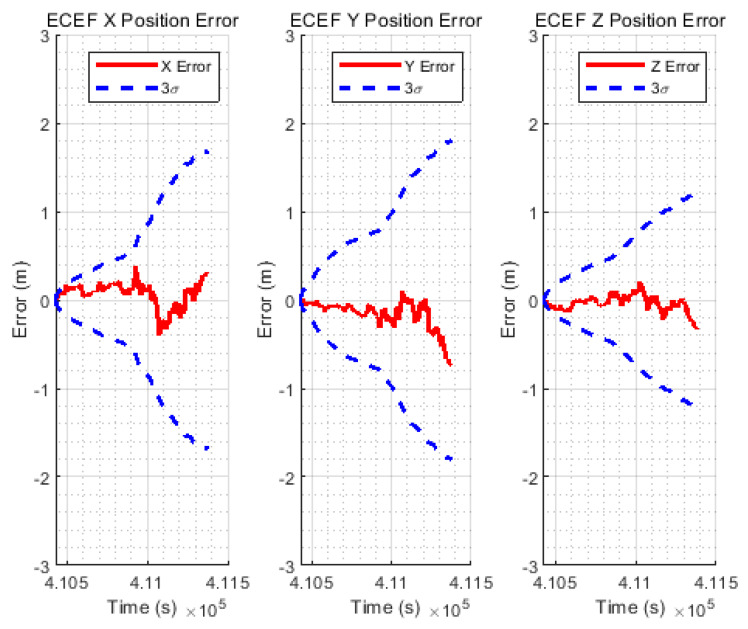
Time history of XYZ errors with 3σ.

Figure 12 shows the time history of estimation errors in the ECEF XYZ coordinate. The dashed blue line indicates the 3σ value estimated by the filter, and the solid red line indicates the positioning error. Over time, the 3σ value accumulates and becomes larger while bounding the actual error of estimation well. 

**Figure 13:**
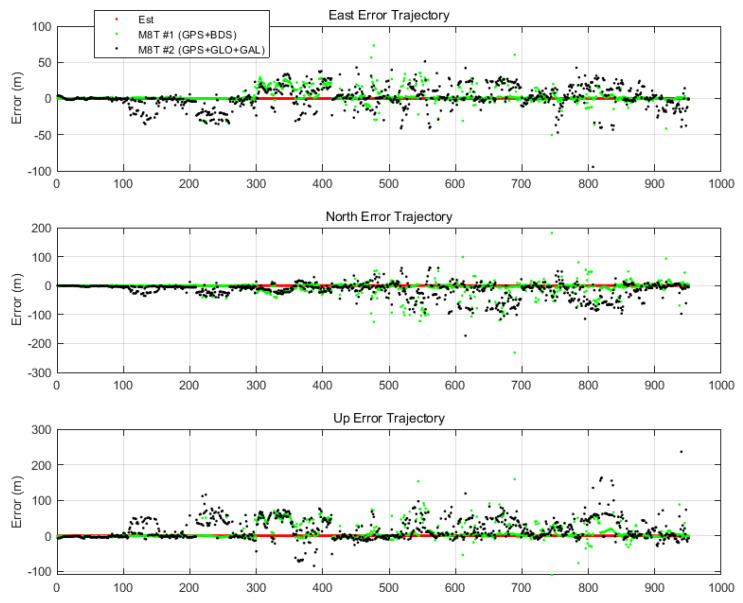
Time history of east–north–up (ENU) errors with standalone positions.

The result obtained by the proposed algorithm was compared with the results of other receivers in east–north–up (ENU) coordinates, as shown Figure 13. We first compared the results to the standalone positions based on pseudorange measurements of two Ublox M8T used in the experiment. 

The red points indicate ENU errors of the proposed TDCP/INS navigation system, the green points indicate errors of the standalone position of M8T #1 (GPS and BeiDou), and the black points indicate errors of the standalone position of M8T #2 (GPS, GLONASS, and Galileo). In the standalone results, it can be identified that severe positioning errors were caused by multipath errors. On the other hand, the proposed navigation system had a more robust and stable performance than the standalone results. Numerically, it had a horizontal root-mean-square (RMS) of 17 cm and a maximum positioning error of 43 cm for 15 min. 

**Figure 14:**
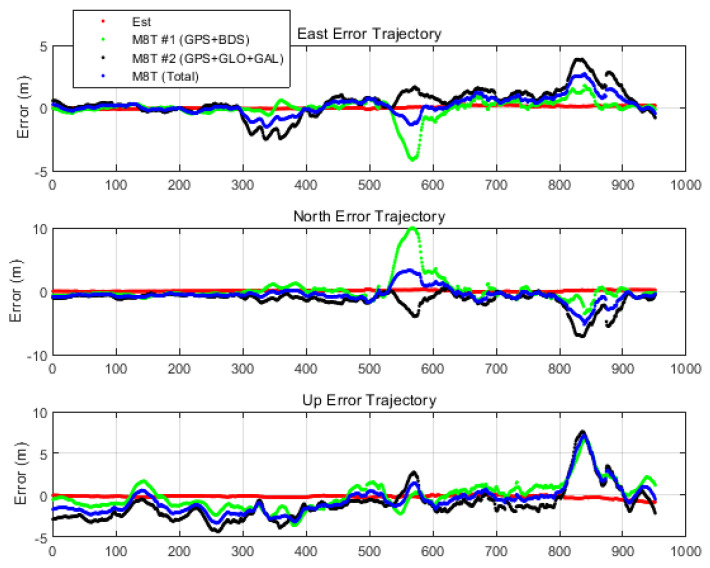
Time history of ENU errors with filtered solutions.

However, most of the low-cost GNSS receivers, like Ublox M8T, have their own filter algorithms, and the final position outputs are a filtered solution which shows a significantly reduced impact of multipath error. Thus, for a more practical comparison of results, the real-time filtered position outputs of two Ublox M8Ts were used, and the result obtained by the proposed algorithm was also compared in the east–north–up (ENU) coordinate, as shown in Figure 14. The red points indicate ENU errors in the proposed TDCP/INS navigation system, the green points indicate the errors in the real-time position output of M8T #1 (GPS and BeiDou), and the black points indicate the errors in the real-time position output of M8T #2 (GPS, GLONASS, and Galileo). The results of both M8Ts had large positioning errors of up to 5~10 m due to multipath errors in the deep urban environment. The errors in the latter part of the experiment were greater than those in the early part because the land vehicle was driving towards a more urban area. On the other hand, the estimated position through the proposed algorithm had a smaller error level compared with others. Furthermore, the result of the average of the two Ublox outputs was also plotted for equal comparison with the result of the proposed system using all the constellation’s measurements. It is confirmed that it could not have sub-meter level accuracy due to the multipath error. 

**Figure 15:**
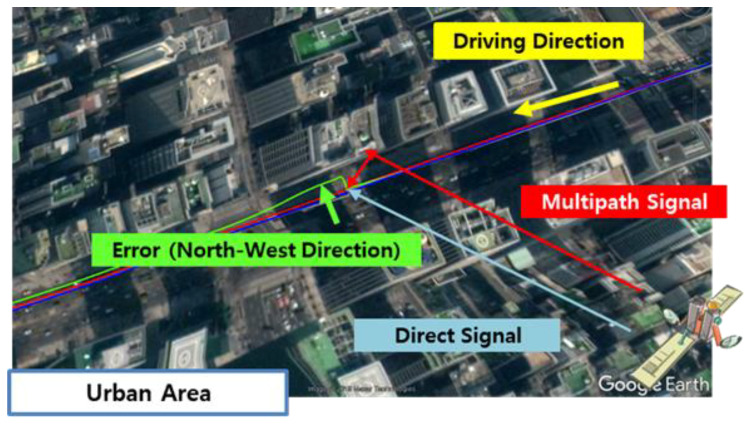
Horizontal trajectory on Google earth.

Figure 15 shows the trajectories from 500 to 600 s on Google Earth. As shown in the figure, the buildings were located in the north area of the land vehicle driving on Teheran road, and it is predicted that the large errors in the northwest direction occurred due to the reflected signals by the buildings located in the north area.

Table 1 and Table 2 list the detailed numerical results of the dynamic test with error variables such as RMS and maximum. As shown in Table 1, we can see that the proposed TDCP/INS navigation algorithm in this study was the most accurate and had the smallest error of less than 20 cm. In addition, the RMS of other methods were successful with accuracy at the 1~2 m level. However, considering the safety of autonomous vehicles, the most important factor is being able to obtain a precise position continuously. If a serious position error over 10 m occurs in an instant, the autonomous vehicle may cause an accident. Therefore, attention should be paid to the maximum error rather than the average accuracy such as RMS. Table 2 shows the maximum errors, and the proposed navigation method had a more accurate performance than others. Ublox had an extremely large level of error, which cannot ensure the safety of users. 

**Figure 16:**
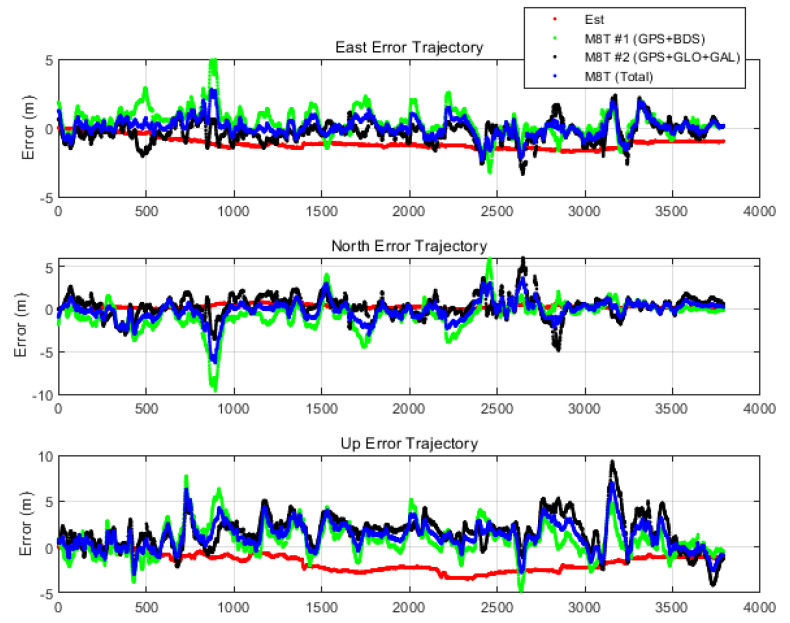
Time history of ENU errors with filtered solutions (1 h).

To verify the performance of the proposed system over an increased time period, a secondary urban real test was carried out for 1 h. All equipment and configurations of the test were the same as the previous test. This secondary test was performed on 19 September 2019. It was assumed that only the initial position was known precisely, and any correction information was not used. Figure 16 shows the positioning result of the proposed navigation system and the real-time filtered position outputs of the two Ublox M8Ts in ENU coordinates. 

From the result of the proposed system, it can be observed that the positioning error gradually increased over 1 h. However, the positioning error was less than that of the filtered position outputs of low-cost GPS receivers, which were instantaneously contaminated by the multipath error at several times. The results of both M8Ts had a maximum positioning error of up to 5~10 m due to multipath error in the deep urban environment. On the other hand, the proposed system had a much more robust and precise navigation performance at several points as opposed to both M8Ts. Table 3 and Table 4 list the detailed numerical results of the long-time dynamic test. Filtered solutions had RMS at the 1 m level due to the averaging effect over a long time. However, the maximum error of filter solutions showed the effect of the multipath error. Numerically, the proposed navigation system had a 1.22 m horizontal RMS error and a 2 m maximum error over 1 h. Thus, it was confirmed that it also had a robust positioning performance in terms of multipath error over a long time.

Based on the above results, multipath errors can cause a sudden position jump in navigation solutions of low-cost, single-frequency GPS receivers in extreme urban areas. It may cause safety problems such as vehicle accidents. The limitations of the multipath errors include not only degraded accuracy but also difficult error modeling. Thus, it is also not possible to provide a confidence level for the position solution, and the precise navigation performance cannot be guaranteed. On the other hand, the proposed navigation system can cope with safety threats by providing a confidence level, which can be estimated by the TDCP/INS filter as shown in Figure 12.

## 4. Conclusions

In this study, we developed a new vehicle navigation system for a deep urban multipath environment using the TDCP/INS filter proposed in a previous study [19]. We utilized time differencing on consecutive carrier phase measurements, which are robust to urban multipath errors, to eliminate the need to determine integer ambiguity that is time-invariant. 

To obtain highly precise positioning accuracy and prepare for any failures in measurements, such as cycle slips, the TDCP/INS navigation system was finally constructed using only low-cost sensors in combination with INS. Furthermore, we improved the performance of the cycle slip detection algorithm using multi constellation and ignoring system clock differences.

Finally, the dynamic field tests in a deep urban area were conducted to verify the accuracy of the proposed system. In the tests, it is confirmed that the proposed system can achieve horizontal accuracy of approximately 20 cm over 15 min and about 1 m over 1 h. Thus, we can confidently say that it can be used as a precise navigation system in a deep urban multipath area. On the other hand, in the standalone results, it can be identified that severe positioning errors were caused by the multipath errors. Figure 15 shows how the multipath signal influenced the position errors. The large position errors in the northwest direction occurred due to the reflected signals by the buildings located in the north area. 

In the proposed navigation system based on carrier phase measurements, which are theoretically robust to multipath error, the maximum position error was larger in the test results of extreme urban areas compared to the expected values. This result might be attributed to the multipath error of up to 4.76 cm in the carrier phase measurements with poor dilution of precision (DOP), which occurred due to the small number of visible satellites. In order to prevent error increases due to poor DOP, a barometric altimeter can be utilized. In addition, the proposed system is expected to be able to apply correction information such as RTK and a satellite-based augmentation system (SBAS) to obtain a more precise level of accuracy.

## Figures and Tables

**Table 1:** RMS errors of the proposed method with other filtered solutions.

RMS ^1^	TDCP/INS (Proposed)	Ublox M8T #1	Ublox M8T #2	Ublox M8T #1, #2 Average
East	0.11 m	0.80 m	1.23 m	0.78 m
North	0.13 m	2.03 m	1.87 m	1.31 m
Up	0.24 m	1.67 m	2.31 m	1.85 m

**^1^** Root-mean-square (RMS).

**Table 2:** Maximum errors of the proposed method with other filtered solutions.

RMS ^1^	TDCP/INS (Proposed)	Ublox M8T #1	Ublox M8T #2	Ublox M8T #1, #2 Average
East	0.27 m	4.14 m	3.89 m	2.73 m
North	0.34 m	10.04 m	7.13 m	5.25 m
Up	0.81 m	6.88 m	7.60 m	7.26 m

**^1^** Root-mean-square (RMS).

**Table 3:** RMS errors of the proposed method with other filtered solutions (1 h).

RMS ^1^	TDCP/INS (Proposed)	Ublox M8T #1	Ublox M8T #2	Ublox M8T #1, #2 Average
East	1.18 m	1.06 m	0.82 m	0.68 m
North	0.33 m	1.77 m	1.30 m	1.20 m
Up	1.89 m	1.84 m	2.45 m	1.90 m

**^1^** Root-mean-square (RMS).

**Table 4:** Maximum errors of the proposed method with other filtered solutions (1 h).

RMS ^1^	TDCP/INS (Proposed)	Ublox M8T #1	Ublox M8T #2	Ublox M8T #1, #2 Average
East	1.73 m	4.97 m	3.37 m	2.77 m
North	0.89 m	9.61 m	6.01 m	6.32 m
Up	3.47 m	7.73 m	9.32 m	6.99 m

**^1^** Root-mean-square (RMS).
